# Diversifying the medical workforce requires a step change in widening participation - Putting the cat amongst the pigeons!

**DOI:** 10.15694/mep.2018.0000279.1

**Published:** 2018-12-11

**Authors:** Sandra Nicholson

**Affiliations:** 1 QMUL

**Keywords:** diversity, widening participation, medical workforce, social inclusion, social mobility

## Abstract

This article was migrated. The article was marked as recommended.

Worldwide there is a call for diversifying the healthcare workforce through widening participation (WP) necessitating a significant change in current recruitment and selection policies and practices. However this is advocated without a well understood conceptualisation of WP or consensus between stakeholders about its purposes.

Employers, and patients, require a medical workforce that is both caring and competent but also accessible. This presents a significant challenge for most countries, especially in some areas of healthcare, for both common and varying reasons. For example Australia and NZ have long struggled to recruit healthcare workers to remote and rural parts of their countries, whilst currently general practice within the UK, is in crisis due to under recruitment and low morale.

Higher Education institutions wish to recruit the best students but it is not clear who the “best students” are and who will progress to fulfil future workforce requirements. Globally universities are concerned by market forces and face significant financial constraints often directly competing with WP policies. Furthermore an established culture of meritocracy, schemes to “top-up” perceived deficits within certain student groups, and fears surrounding the performance of non-traditional students once at university, deter both would be students and the institutions themselves from increasing diversity.

Whilst medical schools may aspire to the aims of social justice and fair opportunities such a trajectory is plagued with difficulties, such as university tariff leagues tables, and concerns with student attrition and differential attainment. However considering the aforementioned workforce issues it is timely to look afresh at what our priorities are in selecting for the future medical workforce. Better understanding the available evidence and the inherent tensions within WP would help us make the required step change that facilitates selecting a more appropriate medical workforce.

## What do we mean by diversity?

Diversity is commonly described as aiming to recognise, respect and value people’s differences to contribute and realise their full potential by promoting an inclusive culture. As such diversity is frequently associated with notions of equality, fairness and democracy. However the conceptualisation of diversity and its purported aims, purposes and surrounding discourse within healthcare remain contested. This is highlighted by the presumed relationship between increasing diversity through adopting appropriate widening participation (WP) policies and practices.

Widening participation has been defined by the policy that people such as those coming from disadvantaged backgrounds, mature students, students from ethnic and cultural groups, and disabled students should be encouraged to take part, and be represented proportionately, within higher education (HE). This relates to improving social mobility which is seen as breaking the transmission of disadvantage from one generation to the next ( Nicholson and Cleland, 2015 and Fair Access to Professional Careers, 2012).

Within healthcare themes of ‘diversity’, ‘equality’ and ‘inclusion’ are therefore seen as key forms of rhetoric related to WP within the broader context of defining social justice, social mobility and sustaining a fair selection policy. However much is contested and diversity doesn’t equal equality, and equality doesn’t ensure equity, and whilst both diversity and equality are used as tools for promoting WP consensus between stakeholders is lacking.

“ *Putting the cat amongst the pigeons*” is a British idiom often used to prompt and provoke further critical debate and questioning usually by revealing a controversy. Using this stance I would propose that whilst medicine, and ultimately patient care, may benefit from diversifying the medical workforce, this strategy has a weak evidence base, and has both overt and covert tensions at play, which may additionally undermine its effectiveness. Furthermore using initiatives to widen the participation of medical students from under-represented groups, irrespective of how laudable, has largely failed to achieve the desired diversification of the healthcare workforce (Cleland et al. 2012; Larkins et al. 2015).

Therefore in the following sections I highlight why in my opinion WP is important, and how by exposing the tensions within WP policy and practice, this may facilitate the education community in making the step change required to successfully diversify the healthcare workforce.

## Why does this matter?

It is important that whilst the evidence base is weak there is some evidence that the diversity of the medical workforce improves healthcare, largely based on the concept of “like would treat like” ( James et al., 2008), with increased patient satisfaction ( Laveist et al, 2002; Cooper et al, 2003). However these outcomes are dependent on such students, once qualified, choosing to practice in areas of diversity and deprivation. There is some evidence to suggest that health professional trainees from lower socio-economic backgrounds may be more likely to end up working in lower socio-economic areas at the completion of their training, and are more likely to choose to work in a community setting or psychiatry. This has traction for under-represented specialities such as general practice and psychiatry in the UK, and rural recruitment in countries such as Australia.

To illustrate an example is presented from the University of Western Australia medical school that demonstrates through a successful WP policy increased participation of students from a broader spectrum of the community. This intervention employed expanded selection criteria and a quota-based approach for students of rural, indigenous and other socio-educationally disadvantaged backgrounds. Students who were categorised in the lower 8 socio-economic deciles at entry to medical school had increased odds of a current practice address in the lower 8 socio-economic deciles 5 or more years after graduation (OR 2.05, 95% CI 1.72, 2.45, P < 0.001) ( Puddey, Playford, and Mercer A., 2017). Further research highlights the complexity of this field with more nuanced findings that show that some students from very low socio-economic backgrounds not preferring to work in deprived areas (Griffin, Porfeli and Hu, 2017).

There is a risk that the constructs associated with diversity and WP may be used subconsciously or actively to undermine the enactment of social justice and social mobility if approaches that favour only “diversity economics” are employed ( Archer, 2007). Diversity economics is a means for exploiting pools of talent, such as under-represented socio-economic groups, to increase national and local economic productivity, and as in the case of healthcare provide resources in areas of unmet need. However policies aligned with “diversity economics” are not primarily interested in social justice or social mobility but have been likened more with social control ( Archer, 2007). This highlights some of the possible tensions driving WP and diversification of the healthcare workforce that its members or prospective members may not be aware of.

## Has WP failed?

Many have lamented the lack of progress of WP in the UK and some like the author of the quote below indicating that the blame lies with the medical profession itself:

“ *Medicine .. has a long way to go when it comes to making access fairer, diversifying its workforce and raising social mobility.. Its success in recruiting more female doctors and doctors from black and minority ethnic backgrounds indicates that with the right level of intentionality the medical profession can also throw open its doors to a far broader social intake than it does at present. .. Overall, medicine has made far too little progress and shown far too little interest in the issue of fair access. It needs a step change in approach*” (Fair Access to Professional Careers, 2012, p3).

However whilst significant under-representation of some social, cultural and ethnic groups in medical schools and medicine worldwide persists despite a variety of national initiatives (e.g. quota systems, political imperatives) and local activities (e.g. pipeline programmes) to ameliorate such under-representation the reasons for this disappointing result remain elusive ( GMC, 2013, Mathers et al. 2011 and Southgate, 2015).

One of the most alarming issues is that despite high intensity interaction and expensive resourcing very little is known about what actually works with WP and in what context. The evidence on whether or not selection practices support increasing diversity to medicine is sometimes conflicting. For example, Tiffin et al. (2012) found that certain ways of using the UKCAT (an admissions test) were associated with a higher proportion of students from under-represented groups being admitted to UK medical schools whilst another longitudinal study, Mathers and colleagues, 2011, failed to identify any consistent effect of different usages of the UKCAT on equity in selection processes. What is clear is that more good quality research, some of which needs to be longitudinal, is desperately required and initiatives such as the UKCAT and UKMED databases that facilitate such research are to be applauded.

One such innovative study examined if changes in medical school selection criteria or processes impacted on the demographic composition of the student population. This was an observational study of medical students from 18 UK 5-year medical programmes who took the UKCAT from 2007-2014. The authors concluded that although schools changed their selection procedures, these changes did not lead to any observable differences in their student populations ( Fielding et al, 2018).

Sometimes WP initiatives fail to diversify because medical schools take different positions in relation to interpreting and translating WP policy. Further exploration of what happens when policy enters practice is neglected in medical education. Two studies that did examine this confirm the earlier assertion that WP is contested, and because of this medical schools implemented WP policy variably. For example despite available evidence UK medical schools are reluctant to use contextual admissions despite WP charities calling for radical changes in the use of markers of social and educational disadvantage ( Cleland, Nicholson, Kelly and Moffat (2015).

## What might make a step difference to WP?

It is clear that heavily weighted usage of prior academic attainment is a common selection screen that presents a hurdle for some students from disadvantaged educational backgrounds that cannot be jumped. This is unfortunate as there is increasing evidence that academic entry requirements can be lowered without admitting students who will academically struggle (HEFCE, 2014). The use of appropriate evidenced based contextual markers can aid careful selection. In addition the combination, weighting and sequencing of selection methods may facilitate changing the socio-economic make up of those selected ( Larkins et al, 2015 and Girotti, Park and Tekian, 2015). Significant interaction from policy makers with those who are to implement policy is required to alleviate anxieties and tensions and provide information surrounding enacting WP policy.

## Conclusions

Overall it can be seen that there remains a contested understanding of WP, its aims, purposes and what constitutes best practice and how medical schools are to effectively implement it. There is unfounded significant anxiety that students from under-represented groups and lower socio-economic backgrounds will require additional labour intensive support and may academically struggle. There is little acknowledgement of what positive attributes these candidates bring to medical school alongside the potential benefits of diversifying the healthcare workforce.

## Take Home Messages

• There remains significant under-representation of some social, cultural and ethnic groups in medical schools and medicine worldwide persists despite a variety of national initiatives.
• Diversifying the healthcare workforce through widening participation (WP) will necessitate a significant change in current recruitment and selection policies and practices.
• Widening participation is a contested field without consensus between stakeholders about its purposes or best practice in implementation.
• Better understanding the available evidence and the inherent tensions within WP would help us make the required step change that facilitates selecting a more appropriate medical workforce.

## Notes On Contributors

Sandra Nicholson is Professor of Medical Education at Barts. She is the Deputy Director of the Institute for Health Sciences and Head of the Centre for Medical Education. She continues to practise as a GP and is passionate about WP.

## References

[ref1] Archer L. (2007). Diversity, equality and higher education: a critical reflection on the abuses of equity discourse within widening participation. Teaching in Higher Education. 12:5-6,635–653. 10.1080/13562510701595325

[ref2] Cooper L.A. Roter D.L. Johnson R.L. Ford D.E. Steinwachs D.M. (2003). Patient-centered communication, ratings of care, and concordance of patient and physician race. Annals of Internal Medicine. 139,907–915. 10.7326/0003-4819-139-11-200312020-00009 14644893

[ref3] Cleland J. A. Nicholson S. Kelly N. & Moffat M. (2015). Taking context seriously: Explaining widening access policy enactments in UK medical schools. Medical Education. 49(1),25–35. 10.1111/medu.12502 25545571

[ref4] Cleland J. Nicholson S. Patterson F. Thomas E. Wilde K. , (2017). Exploring Admissions Deans’ views of the use of contextual data in UK medical school selection processes. Paper presented at Association Medical Education in Europe.

[ref5] Fielding S. Tiffin PA. Greatrix R. Lee AJ. Patterson F. , (2018). Do changing medical admissions practices in the UK impact on who is admitted? An interrupted time series analysis. BMJ Open. 8(10):e023274. 10.1136/bmjopen-2018-023274 PMC6194474 30297349

[ref6] General Medical Council (GMC) . National training survey 2013: socioeconomic status questions. 2013; Available at: https://www.gmc-uk.org/-/media/documents/Report_NTS_Socioeconomic_Status_Questions.pdf_53743451.pdf Accessed March, 2018.

[ref7] Girotti J. A. Park Y. S. & Tekian A. (2015). Ensuring a fair and equitable selection of students to serve society’s health care needs. Medical Education. 49(1),84–92. 10.1111/medu.12506 25545576

[ref8] HEFCE , (2014). Differences in degree outcomes. The effect of Student and subject characteristics.

[ref9] James D. Ferguson E. Powis D. Symonds I. & Yates J. (2008). Graduate entry to medicine: widening academic and socio-demographic access. Medical Education. 42(3),294–300. 10.1111/j.1365-2923.2008.03006.x 18275417

[ref10] Larkins S Michielsen K Iputo J Elsanousi S Mammen M , (2015). Impact of selection strategies on representation of underserved populations and intention to practise: international findings. Med Educ. 49(1):60–72. 10.1111/medu.12518 25545574

[ref11] Laveist T.A. & Nuru-Jeter A. (2002). Is doctor-patient race concordance associated with greater satisfaction with care? Journal of Health and Social Behavior. 43,296–306. 10.2307/3090205 12467254

[ref12] Mathers J. Sitch A. Marsh J.L. & Parry J. (2011). Widening access to medical education for under-represented socioeconomic groups: population based cross sectional analysis of UK data’, 2002-6. BMJ. 342,918–918. 10.1136/bmj.d918 PMC3043108 21343208

[ref13] Milburn A. (2012). Fair Access to Professional Careers. A progress report by the Independent Reviewer on Social Mobility and Child Poverty.

[ref14] Nicholson S. & Cleland J. (2015). Reframing research on widening participation in medical education: using theory to inform practice.In Cleland J. & Durning S. J. (Eds.), Researching medical education.(pp.231–243). Wiley: UK. 10.1002/9781118838983.ch20

[ref15] Puddey IB Playford D Mercer A . (2017). Impact of medical student origins on the likelihood of ultimately practicing in areas of low vs high socio-economic status. BMC Medical Education. 17(1):1–13. 10.1186/s12909-016-0842-7 28056975 PMC5215143

[ref16] Southgate E. Kelly B. J. & Symonds I. M. (2015). Disadvantage and the ‘capacity to aspire’ to medical school. Medical Education’. 49(1),73–83. 10.1111/medu.12540 25545575

[ref17] Tiffin P.A. Dowell J.S. & McLachlan J.C. (2012). Widening access to UK medical education forunder-represented socioeconomic groups: modelling the impact of the UKCAT in the 2009 cohort. BMJ. 344,e1805. 10.1136/bmj.e1805 22511300 PMC3328544

