# Supplementary material for: Diversifying the medical workforce requires a step change in widening participation - Putting the cat amongst the pigeons!
Source: MedEdPublish (2016). 2018 Dec 11;7:279. [Version 1] doi: 10.15694/mep.2018.0000279.1 (PMC10711969; doi:10.15694/mep.2018.0000279.1)
Supplement: Peer review history

# Reviewer report: Reviewer response for version 1

**DOI:** 10.21956/mep.19736.r29575

**Published:** 2020-03-03

**Authors:** Sarmishtha Ghosh

**Affiliations:** 1 International Medical University

This review has been migrated. The reviewer awarded 4 stars out of 5 The article is a personal opinion of prof Sandra. She has mentioned very clearly the purpose of the article as she found gaps in the process. The writing is very lucid explanative and substantiated by evidence from relevant resources. However, the term " widening participation" is not very common in every corner of the globe. Even though it is defined categorically, it will be difficult for educators in Asia to understand and appreciate the problem. A reference to countries other than the UK, Australia would have been appreciated. Underrepresentation is dealt with in different ways in different countries. Dr. Sandra's paper can serve as an eye-opener to a very important often neglected area of student selection.As pointed out by Prof. Trevor, this paper is recommended for all those who are associated with selection or making the policies of selection in the medical councils of a country.

Reviewer Expertise:

NA

No decision status is available

# Reviewer report: Reviewer response for version 1

**DOI:** 10.21956/mep.19736.r29574

**Published:** 2019-08-26

**Authors:** Alaa Dafallah

**Affiliations:** 1 University of Khartoum

This review has been migrated. The reviewer awarded 4 stars out of 5 The author addresses a key issue, relevant to medical education and reflects her opinion in a very critical and holistic approach stimulating much debate and uncovering gaps that need to be filled. However, I feel the abstract could be more bold in reflecting the authors key messages and perspective. I also wish to draw attention to the fact that reaching a consensus on best practice in widening participation might not be attainable, because in my humble opinion, what determines the effectivity of WP strategies is the context. Different factors such as community demographics, social & cultural norms, public policies etc influence WP and hence what works in the UK will not be what will work in a country like Nigeria for example. How wide differs for different contexts. I would also like to highlight that whilst widening participation is perceived largely as a mechanism to bridge the gap in accessibility to health workforce, but also inequity in education. There is a need to define how “wide”. Under-represented groups ? marginalized groups too ? Also there is need for more research to uncover data on the status qo of representation of social, cultural or ethnic groups in medical schools, justifying the authors claim.

Reviewer Expertise:

NA

No decision status is available

# Reviewer report: Reviewer response for version 1

**DOI:** 10.21956/mep.19736.r29573

**Published:** 2019-06-10

**Authors:** Trevor Gibbs

**Affiliations:** 1 AMEE

This review has been migrated. The reviewer awarded 4 stars out of 5 An interesting paper that covers a difficult subject but one that, as the author points out, is under inverstigated. I thought that this was a very well written paper that asked more questions rather than provide answers, so I would recommend it to all those involved in selection processes.

Reviewer Expertise:

NA

No decision status is available

# Reviewer report: Reviewer response for version 1

**DOI:** 10.21956/mep.19736.r29577

**Published:** 2019-01-10

**Authors:** Sally Curtis

**Affiliations:** 1 Southampton medical school

This review has been migrated. The reviewer awarded 5 stars out of 5 Professor Nicholson’s views are very important and helpful in opening up the much-needed wider debate around widening participation in medicine. This opinion piece highlights some of the tensions between the drive to increase participation of underrepresented groups in medicine and the societal and educational barriers that still exist. Undoubtedly, there is a need for more research to provide evidence of the effectiveness of WP initiatives in admissions and in student progression and retention. In addition, evidence to demonstrate the value of widening participation to the profession and to the undergraduate education experience would provide new and important perspectives. Although to date the changes in numbers of medical students from underrepresented are small, they are increasing. As highlighted in this article, more evidence and will help provide a wider, supportive framework to optimise meaningful success of widening participation to medicine.

Reviewer Expertise:

NA

No decision status is available

# Reviewer report: Reviewer response for version 1

**DOI:** 10.21956/mep.19736.r29576

**Published:** 2018-12-28

**Authors:** Annette Mercer

**Affiliations:** 1 UWA

This review has been migrated. The reviewer awarded 4 stars out of 5 This opinion piece provides an important approach to an area which is not well-understood and indeed, is not well-supported by some members of the medical education community. The paper provides a start on unpicking a delicate and problematic area related to the philosophy of selection as well as the application of the principles of selection to achieve WP outcomes. I believe that this opinion piece is valuable in attempting to put these issues into the spotlight and to generate dialogue on a new approach to the selection of medical students as well as an investigation of the most practical methods of achieving a more diversified medical workforce. Such dialogue should most appropriately be undertaken in a global context, given the mobility of members of the medical profession and the benefits which technology provides.

Reviewer Expertise:

NA

No decision status is available
